# Cannabis tolerance reduces symptom relief

**DOI:** 10.3389/fphar.2025.1496232

**Published:** 2025-06-19

**Authors:** Sarah S. Stith, Xiaoxue Li, Franco Brockelman, Keenan Keeling, Branden Hall, Jacob M. Vigil

**Affiliations:** ^1^ Department of Economics, University of New Mexico, Albuquerque, NM, United States; ^2^ MoreBetter, Ltd., Hyattsville, MD, United States; ^3^ Department of Psychology, University of New Mexico, Albuquerque, NM, United States

**Keywords:** tolerance, cannabis, marijuana, addiction, cannabidiol, tetrahydrocannabinol, cannabis chemovars, complementary medicine

## Abstract

**Objectives:**

We measure for the first time how tolerance from repeated consumption of medical cannabis affects acute symptom management.

**Methods:**

Using the Releaf App, medical cannabis patients recorded their symptoms, product type, cannabis consumption method, major cannabinoid levels, dosing patterns, and real-time symptom intensity levels prior to and following each cannabis administration session, as well as any side effects from usage. The sample consists of the first ten cannabis self-administration sessions recorded by 16,395 medical cannabis patients between 06/05/2016 and 09/19/22, yielding a sample of 120,691 symptom-specific treatment-level observations, recorded during 42,005 sessions. This study uses fixed effects least-squares regression analyses to analyze the effects of the session count on symptom relief.

**Results:**

On average, people experienced a 0.5% decrease in symptom relief with each subsequent session (p < 0.001). Combustible products offered more therapeutic relief than vaping, eating or drinking; higher doses offered greater relief; and the reduction in symptom relief with subsequent usage was similar whether patients were treating pain, depression, or anxiety. Cannabis products’ THC levels were positively associated with symptom relief; however, patients showed no changes in the THC levels of products with subsequent consumption. Patients increased the dose consumed as they completed more sessions. The results are robust to alternative treatment measures, including days since the first session was recorded. Subsample regressions indicate that experienced users drive most of the effects. Analyses assessing side effects show that factors, such as THC and dose, that increased symptom relief also increased side effects experienced.

**Conclusion and implications:**

The findings suggest the majority of patients experience decreased symptom relief after repeated use of medical cannabis, counterbalanced by improvements in negative side effects. Of direct clinical relevance, THC levels and the dose can be adjusted to customize medical cannabis patient treatment, increase medication compliance, and improve treatment outcomes.

## Introduction

The pharmacodynamics of tolerance to the effects of consuming the *Cannabis* plant is not fully understood. The concept of ‘tolerance,’ or lessening of response to a substance after repeated usage has been primarily and historically referenced within the addiction literature as one among several hallmark features of substance-related pathologies (e.g., cravings, withdrawal), such as *cannabis use disorder* (Kesner and Lovinger, 2021). However, unlike consumption of other naturally addictive substances like caffeine (C8H10N4O2), ethanol alcohol (C2H5OH), morphine (C17H19NO3) and codeine (C18H21NO3), the *Cannabis* plant is comprised of a multitude of phytochemical molecules, including subcannabinoids, terpenes, and flavonoids, that may act additively and/or interactively to produce a wide range of product-specific alterations in cognitive and behavioral functioning (McPartland and Russo, 2001; Vigil et al., 2023; Stith et al., 2018). That is, while cannabis is similar to other addictive substances in generally requiring increased dosing in order to experience similar cognitive and visceral effects across subsequent consumption sessions (Colizzi and Bhattacharyya, 2018; Sim-Selley et al., 2006), the effects of using cannabis can vary widely across products (Stith et al., 2018; Stith et al., 2019). Cannabis is also different from many other addictive substances that have a linear relationship with their acute effects; whereby, the greater the volume of the substance consumed within a single consumption session, the greater the level of objective and subjective intoxication. In contrast, laboratory studies have shown that cannabis’ effects can become blunted, or what may be thought of as product-specific saturation or desensitization effects, within a short-time frame, irrespective of continued consumption (Colizzi and Bhattacharyya, 2018; Piscura et al., 2023), rendering unique aspects of cannabis tolerance effects relative to other common and intoxicating substances.

There are many limitations in the extant research on the effects of using cannabis. Randomized controlled clinical trials often fail to provide generalizable information for patients facing the vast breath of cannabis products currently available recreationally and/or medically due to their focus on synthetic cannabinoids, specific methods of consumption, or a limited number of government-provided cannabis products that can vary greatly in quality and potency from commercially available cannabis products (Stith and Vigil, 2016; National Academies of Sciences E and M, 2017). Individual differences in the effects of consuming cannabis are also common. For example, the half-life of two of the main cannabinoids in the plant can range from a matter of minutes to several days for cannabidiol (CBD) and from 1 day to several weeks for tetrahydrocannabinol (THC) (Millar et al., 2018; Mørland and Bramness, 2020; Huestis et al., 1992). This is due in part to individual differences in mental, behavioral, and physical factors, including body structures, e.g., the timing of peak cannabinoid concentration levels differs depending the volumes of different types of body structures such as adipose tissue (Borgelt et al., 2013; Ashton, 2001; Huestis, 2007; Atakan, 2012). Previous clinical studies also have been limited by the inability to adequately compare across common routes of administration (e.g., smoking versus eating), naturalistic dosing behaviors, and the range of chemotypic properties found among *Cannabis* plant strains (Vigil et al., 2023; Andre et al., 2016). The artificial environment created by clinical trials also limits their generalizability, because even basic characteristics of the researchers themselves have been shown to influence the expression of complex forms of health functioning and visceral experience, such as pain percepts (Vigil and Coulombe, 2011; Vigil and Stregnth, 2014; Vigil et al., 2015; Vigil et al., 2014; Vigil and Alcock, 2014). To summarize, the cannabis product and the manner in which it is received through a clinical trial bear little resemblance to cannabis products home-cultivated or purchased from a dispensary budtender (Stith and Vigil, 2016; National Academies of Sciences E and M, 2017). (Many medical cannabis markets also allow designated caregivers to cultivate and purchase cannabis on behalf of a limited number of patients).

Because of these inherent limitations of conducting randomized controlled clinical trials, naturalistic, observational research offers a viable alternative approach for studying how acute and long-term cannabis tolerance may affect health outcomes in people that use the plant medicinally. Observational data that include the wide range of commercially available cannabis products along with the cannabis consumer’s chosen consumption method increase the generalizability of findings and provide practical and clinically relevant information to patients and health providers seeking to optimize cannabis treatment. The urgency of such naturalistic research on cannabis is underscored by the increasing numbers of cannabis users in the United States and beyond, particularly among older adults and people experiencing significant health conditions (Han and Palamar, 2020; Wolfe et al., 2023; Tavabi et al., 2023; Rhee and Rosenheck, 2023).

The current study measures how repeated consumption of common and commercially available medical cannabis products affects patients’ acute symptom management over time using one of the largest samples of real-time, cannabis-consumption sessions in the U.S. This database is collected by the mobile software educational application Releaf App (Releaf App, 2019), which allows users to track their product characteristics, dosing patterns and symptom relief in real time. In the current study, we focus on the roles of repeated use, assessing and controlling for the effects of routes of administration, major cannabinoid contents of the products consumed, reported dosage behaviors, and individual characteristics, including user experience levels and discrete medical conditions being treated.

## Methods

### Data and sample

The Releaf App, a commercial mobile software application designed for patients to measure the effects of using cannabis was used in the current study. The app enables patients to track symptom relief across products types, consumption methods, product potency, dosages, symptoms, and over time. We obtained these data subject to a data use agreement between the University of New Mexico and the owner of the Releaf App, MoreBetter, Ltd. The study was deemed exempt from Institutional Review Board review by the Institutional Review Board at the University of New Mexico.

Users of the app voluntarily download it from either the Google or Apple app store. No compensation is offered to users, e.g., for completing sessions. To start recording a session, users must first record the characteristics of the product, including product type (flower, concentrate, edible, topical, pill, or tincture), and combustion method (joint, pipe, or vaporizer). Plant phenotype (*C. sativa*, *C. indica*, or hybrid) and THC and CBD potency levels are not required reporting but are easy to add during the session setup. The plant phenotype and product potency are usually available on the package of the product, if the product comes from a dispensary. While the plant phenotype of home-cultivated or products obtained through caregiver cultivations may be known, the THC and CBD potencies are only available through laboratory testing, which is costly and only mandated for dispensary-sourced products. Once a product is recorded, it is saved under the user profile and is available for selection in future sessions. After recording the product characteristics or selecting a saved product, the user is required to select the symptom(s) being treated from a list of possible symptoms. Fifty-six possible symptoms are included, with the most commonly reported symptoms related to pain, depression, and anxiety or stress. All symptoms reflect a negative condition except for “Wellness,” which we do not include in the sample, as one cannot treat “Wellness” in a comparable way to how one treats pain or other negative conditions. For each chosen symptom, the user records their initial symptom intensity level on an analog scale from 0 to 10. They are then directed to use the cannabis product and encouraged to record each time they consume a dose, e.g., an inhalation from a vaporizer or a square of chocolate. While the session is active, users can update the symptom intensity level at any time and select any side effects they experience from a list of possible side effects. If a user treats multiple symptoms in one session, the symptom intensity levels are recorded separately for each symptom while the side effects are recorded at the overall session level. Finally, the user ends the session, and no further action is recorded.

**FIGURE 1:**
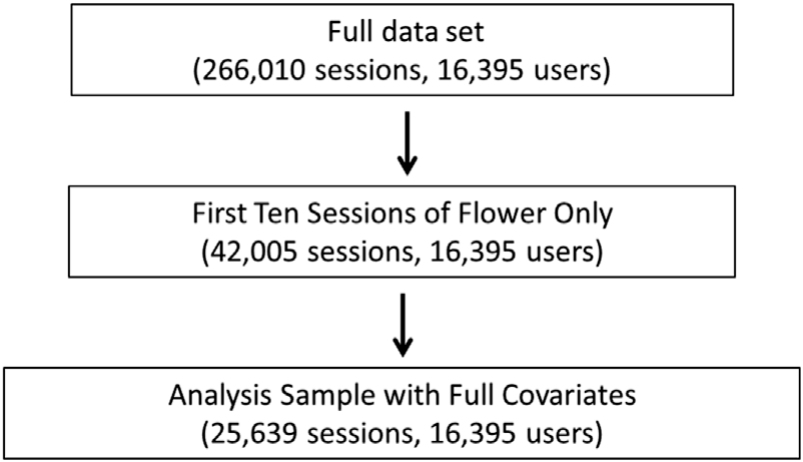
Flowchart of exclusionary criteria for data analyses.

The inclusion criteria for data analyses are shown in Figure 1. To construct the analysis sample, we use symptom-sessions administered between 6/5/2016 and 9/19/2022 that had a starting symptom level greater than zero and had at least one symptom level update recorded within 4 h of the session inception. This sample has 266,010 symptom-specific treatment-level observations recorded in 91,638 cannabis administration sessions, conducted by 16,395 users. App users completed an average of 7.59 sessions during the study period, with the most frequent user recording 912 sessions. To avoid the concern that our statistical analysis might be driven largely by the most frequent app users, we restrict the analysis sample to the first 10 sessions conducted by a user. Further, we exclude sessions in which the user reported using a topical product due to the relative infrequency of this form of cannabis administration in the data. The resulting sample consists of 120,691 symptoms treated in 42,005 sessions by 16,395 users. The analysis sample varies depending on the variables included from the full sample of 120,691 symptom-specific treatment-level observations to 25,639 in the sample including all covariates. The largest reduction comes from including THC and CBD percentages, which are not required variables for completing a session, are only reported on laboratory-tested products, do not add value to the individual user, and are not available for products measuring THC and CBD in milligrams, such as “edibles”. We are unable to convert milligrams of THC and CBD into percentages for comparability with other products because the total product weight is not a variable collected by the Releaf App.

### Variables

Our main treatment variable is the session count from 1 to 10. We assign the user-specific session count chronologically based on session start time.

The primary outcome of interest is symptom change. We measure symptom change as the lowest symptom intensity level during the session minus the starting symptom level, i.e., the maximum level of symptom relief reported. A negative value for the symptom change variable implies a decrease in symptom intensity. As shown in Table 1, on average, users reported a an average starting symptom level of 5.91 and a minimum symptom level of 2.13, indicating an average symptom change of −3.78.

**TABLE 1:** Summary statistics.

	N	Mean	Std. Dev	Min	Max
Product characteristics and symptom information (measured at session-symptom level)					
Smoke (joint or pipe)	115,208	0.45	0.50	0	1
Vape	115,208	0.40	0.49	0	1
Eat or drink	115,208	0.15	0.35	0	1
Product THC	49,022	36.69	27.90	0	100
Product CBD	30,800	11.16	16.27	0	100
Total reported dose	120,586	6.61	9.45	1	785
Starting symptom level	120,691	5.92	2.24	1	10
Minimum symptom level	120,691	2.13	2.00	0	10
Symptom change	120,691	−3.78	2.39	−10	0
Days since first session	193,744	16.50	22.02	0	90
Side effects (measured at session level)					
Share negative	42,005	0.12	0.13	0	1
Share positive	42,005	0.24	0.18	0	1
Share context specific	42,005	0.20	0.18	0	1
User experience (measured at user level)					
Experienced	10,454	0.54	0.50	0	1

Notes: Sample is restricted to sessions with a starting symptom level greater than zero and at least one symptom update within 4 hours.

For covariates, we construct additional variables for product and session characteristics. We construct a categorical variable that measures the mode of consumption: vape (40%), eat or drink (14.8%), and joint or pipe (45.2%). For concentrate and flower products, users are required to record their mode of consumption. For edible, pill, and tincture products, the product type is synonymous with the consumption mode, so such sessions are categorized as “eat or drink.” THC and CBD percentages are measured as continuous variables with averages of 36.7 and 11.16 percent respectively. In addition, we measure the total volume consumed by the natural logarithm of total dosage recorded. The dose is reported in almost all sessions, but because dose is not required reporting and requires repeated interaction with the app, this variable may be subject to greater measurement error than some of the other variables. The dose variable does include significant outliers making the natural log a more reliable measure for understanding the average effects. Furthermore, the fact that the effects of cannabis become blunted with continued use rather than leading to ever higher levels of intoxication, as with alcohol, suggests a logarithmic functional form is more appropriate than a linear functional form. We conduct subsample analyses with subsamples defined by the three most commonly reported symptom categories (pain, depression, and conditions related to anxiety, agitation, irritability and stress) and by user experience level, as this may fundamentally affect tolerance. Pain includes all pain-related conditions in the sample^1^ and accounts for 26.2% of observations; depression accounts for 8.8%%; and anxiety-related conditions, which include agitation/irritability, anxiety and stress, account for 30.2%. To measure pre-app cannabis experience, we use user-level reported experience: “Beginner,” “A Little,” “A Lot,” or “Expert.” This variable is recorded at the user level, typically when the user first sets up their account. Of the 16,395 users, 10,454 responded to this question and 54.4% of them self-reported being an experienced user (“A Lot” or “Expert.”).

In extensions of our main analysis, we also assess the effect of session count on THC, CBD and the natural log of the number of doses, which patients may use to mitigate the effects of tolerance, using these as outcome variables.

Our final analyses assess the effect of session count on side effect reporting, with adverse reactions particularly of interest from a clinical perspective. To generate the side effect variables, side effects first were sorted into seventeen adverse, or negative side effects, nineteen beneficial, or positive side effects, and eleven side effects that are context-specific (Supplementary Table S1 reports the prevalence of each side effect and its category.). After categorizing the side effects, we constructed three variables measuring the share of adverse/negative side effects selected, the share of beneficial/positive side effects selected, and the share of context-specific side effects selected. In an average session, users reported 11.7% of the negative side effects, 24.4% of the positive side effects, and 20.1% of the context specific side effects.

### Statistical analysis

The objective of the study is to explore whether patients build up a tolerance to medical cannabis. Specifically, we examine whether the effect of medical cannabis on symptom relief decreases as the user completes more cannabis sessions. To establish this, we conduct baseline regression analyses using symptom relief as the outcome and the user-specific session count (1–10) as the key explanatory variable. We continue to add controls in order of their effects on the sample size, first adding mode of consumption with vape as the base category, before adding THC and CBD. Lastly, due to concerns about measurement reliability, we include the dose variable to control for higher intensity consumption, which, along with THC, could accelerate tolerance or could be used to mitigate the effects of tolerance. We include starting symptom level throughout as a covariate because a higher starting symptom level is mechanically correlated with greater potential symptom relief.

We conduct robustness checks on the session count treatment measure in four ways. First, we relax the assumption that session count has a linear effect on the outcome variables by creating a {0,1} dummy variable for each session count. This allows each session count to have a different effect rather than forcing the change from the first to the second session to be the same as that from the ninth to the 10th. Second, the session count variable may not accurately capture short-term changes in the outcome variables if sessions are entered sporadically or over a long time period, so we conduct additional regressions using the number of days since the first session was recorded in place of the session count and restricting the sample to sessions completed within the first 90 days of app use. Third, we conduct an even more restrictive check on the role of the time period within which sessions are completed by running additional subsample analyses of patients who completed their first ten sessions within 90, 60, 40, 20 and 15 days. Lastly, because more experienced cannabis consumers are hypothesized to exhibit greater tolerance effects, we conduct subsample analyses for more and less experienced cannabis consumers.

Extending the main analysis to explore mechanisms that affect tolerance, THC, CBD, and the natural log of dose are used as outcome variables to capture the extent to which users are attempting to mitigate tolerance effects through increasing product potencies or the volume of cannabis consumed. These regressions do not include the mode of consumption, dose or starting symptom level.

The final set of primary outcomes we assess is side effect reporting. We run separate regressions using the share of negative, share of positive, and share of context-specific side effects as outcome variables. For each outcome, we run two regressions, one including only session count and starting symptom as covariates, the other including the full set of covariates.

Due to the reduction in our sample size from missing THC information, we run regressions using a {0,1} variable for whether THC is missing as an outcome to assess whether THC information systematically differs with session count.

In all models, the analyses approximate within-user effects by including user-level fixed effects to control for factors that are constant within users during our sample period, e.g., gender and race. Standard errors are clustered at the user level to account for heteroskedasticity and within-user correlation. Analyses were conducted using Stata 15.1.

## Results

### Main analyses

Table 2 reports results showing the association between session count and symptom relief. Column 1 uses the model that does not control for product characteristics or the natural log of. Each additional session by the user was associated with a 0.019 percentage point increase in symptom change (p < 0.001), or a 0.5% decrease in symptom relief relative to the average symptom change of −3.78. While the decrease in symptom relief with each additional session may be clinically minimal, assuming a linear pattern, the cumulative effect aggregates to a 5% decrease in symptom relief after ten sessions or a 10% decrease after 20 sessions. If the patient medicates with cannabis daily, the consequences of even small decreases with each administration session become clinically significant within a matter of days or weeks. Column 2 controls for the mode of consumption, using vape as the base category. The results show a similar effect from session count. Relative to vaping, smoking was weakly associated with more symptom relief (p < 0.1) while eating or drinking was associated with less symptom relief (p < 0.01). Columns 3 and 4 further add THC and CBD levels, and the natural log of dose, respectively. The results confirm that additional sessions were associated with a small decline in symptom relief. In addition, THC levels and higher dosage were strong predictors of symptom relief. The mode of consumption was no longer a significant strong predictor of symptom relief. The relative effects of session count and THC percentage suggest that a 4.4 percentage point increase in THC each session would counterbalance the tolerance effect of one additional session. A six percent increase in the dose each session would similarly counterbalance the 0.022-point reduction in symptom relief from one more cannabis consumption session^2^.

**TABLE 2:** The association between session count and symptom change.

	(1) Symptom change	(2) Symptom change	(3) Symptom change	(4) Symptom change
Session count	0.019***	0.017***	0.019***	0.022***
	(0.003)	(0.003)	(0.007)	(0.007)
THC			−0.004***	−0.005***
			(0.001)	(0.001)
CBD			0.001	−0.000
			(0.002)	(0.001)
Smoke (Joint/Pipe)		−0.050*	−0.142	−0.151*
		(0.030)	(0.091)	(0.089)
Eat or Drink		0.104***	0.218	−0.385
		(0.038)	(0.349)	(0.362)
LnDose				−0.386***
				(0.034)
Starting symptom	−0.666***	−0.667***	−0.654***	−0.653***
	(0.004)	(0.004)	(0.008)	(0.008)
Constant	0.086***	0.109***	0.220**	0.868***
	(0.026)	(0.031)	(0.101)	(0.117)
Observations	120,691	115,208	25,640	25,639
R-squared	0.387	0.389	0.387	0.396
Number of users	16,395	15,798	4,787	4,787

Notes: Smoke and eat or drink are relative to vape. Each column represents a separate regression. All regressions include patient-level fixed effects. Standard errors, clustered at the individual patient level, are shown in parentheses. ***p < 0.01, **p < 0.05, *p < 0.10.

**FIGURE 2:**
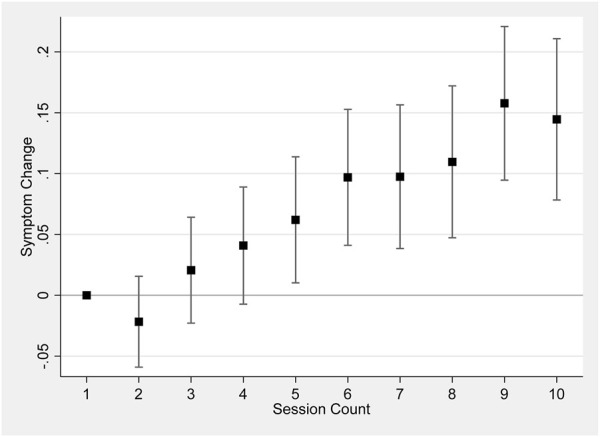
Session count and symptom change. Notes: Figure 2 shows results regressing symptom change on a set of dummy variables for each session count up to 10, including patient-level fixed effects and starting symptom level, and clustering the standard errors at the individual level. 95% confidence bars are shown.

Figure 2 shows the change in symptom relief as the user conducts more sessions, using the first session as the baseline. The model does not control for product characteristics or session dosage in order to maintain a larger sample size (Models controlling for the full set of product characteristics and session dosage are reported in Supplementary Figure S1). The patterns of changes in symptom relief exemplify the findings in Table 1. Later sessions were associated with an increase in symptom change, implying a decrease in symptom relief.

We conduct an additional robustness check on our treatment variable, session count, in which we measure a user’s exposure by the number of days since the user’s first session. We restrict the sample to sessions within 90 days since the first session by the same user. Supplementary Table S2 reports results using this alternative measure. Findings are similar to those in Table 2. Sessions that were conducted on a later date were associated with an increase in symptom change, implying a decrease in symptom relief. The coefficients are smaller than the coefficients for session count, which follows from an average of 3.9 days between recorded sessions. In other words, the coefficients in Supplementary Table S2 reflect the effect of 1 day, while the coefficients in Table 2 reflect the effect of almost 4 days on average.

As a further robustness check on the role of the time period within which sessions are entered, we run regressions restricting the sample to those users who completed their first ten sessions within 90, 60, 40, 20 and 15 days. The coefficients on the session count, reported in Supplementary Table S3, are positive, statistically significant, and range in magnitude from 0.014 to 0.01 as compared with 0.019 in the main sample.

In Table 3, we conduct subsample analyses by pre-app cannabis experience to further explore the process through which tolerance affects symptom relief. Findings suggest that the association between session count and symptom relief was stronger among users who self-identify as experienced users of medical cannabis. For users who were not experienced, session count was significant in the absence of covariates, but not a significant predictor of symptom change once covariates were included. Unfortunately, the sample size is effectively determined by the number of users due to clustering the standard errors, i.e., a lack of power may be behind the statistical insignificance of the still positive coefficient on session count among less experienced users.

**TABLE 3:** The association between session count and side effects.

	(1) Share negative	(2) Share negative	(3)Share positive	(4) Share positive	(5) Share context	(6) Share context
Session count	−0.004***	−0.004***	−0.006***	−0.006***	−0.004***	−0.005***
	(0.000)	(0.001)	(0.000)	(0.001)	(0.000)	(0.001)
THC		0.001***		0.000**		0.001***
		(0.000)		(0.000)		(0.000)
CBD		−0.000		−0.000*		−0.001***
		(0.000)		(0.000)		(0.000)
Smoke (Joint/Pipe)		0.025***		0.036***		0.055***
		(0.008)		(0.011)		(0.010)
Eat or Drink		−0.009		0.040		0.132***
		(0.028)		(0.035)		(0.050)
LnDose		0.016***		0.038***		0.037***
		(0.003)		(0.004)		(0.004)
Starting symptom	0.003***	0.002**	−0.004***	−0.004***	0.000	−0.001
	(0.000)	(0.001)	(0.001)	(0.001)	(0.001)	(0.001)
Constant	0.131***	0.056*	0.266***	0.243***	0.218***	0.233***
	(0.001)	(0.029)	(0.001)	(0.036)	(0.001)	(0.050)
Observations	42,005	9,100	42,005	9,100	42,005	9,100
R-squared	0.013	0.022	0.016	0.043	0.007	0.039
Number of users	13,608	4,118	13,608	4,118	13,608	4,118

Notes: Each column represents a separate regression. All regressions include patient-level fixed effects. Smoke and eat or drink are relative to vape. Standard errors, clustered at the individual patient level, are shown in parentheses. ***p < 0.01, **p < 0.05, *p < 0.10.

**FIGURE 3:**
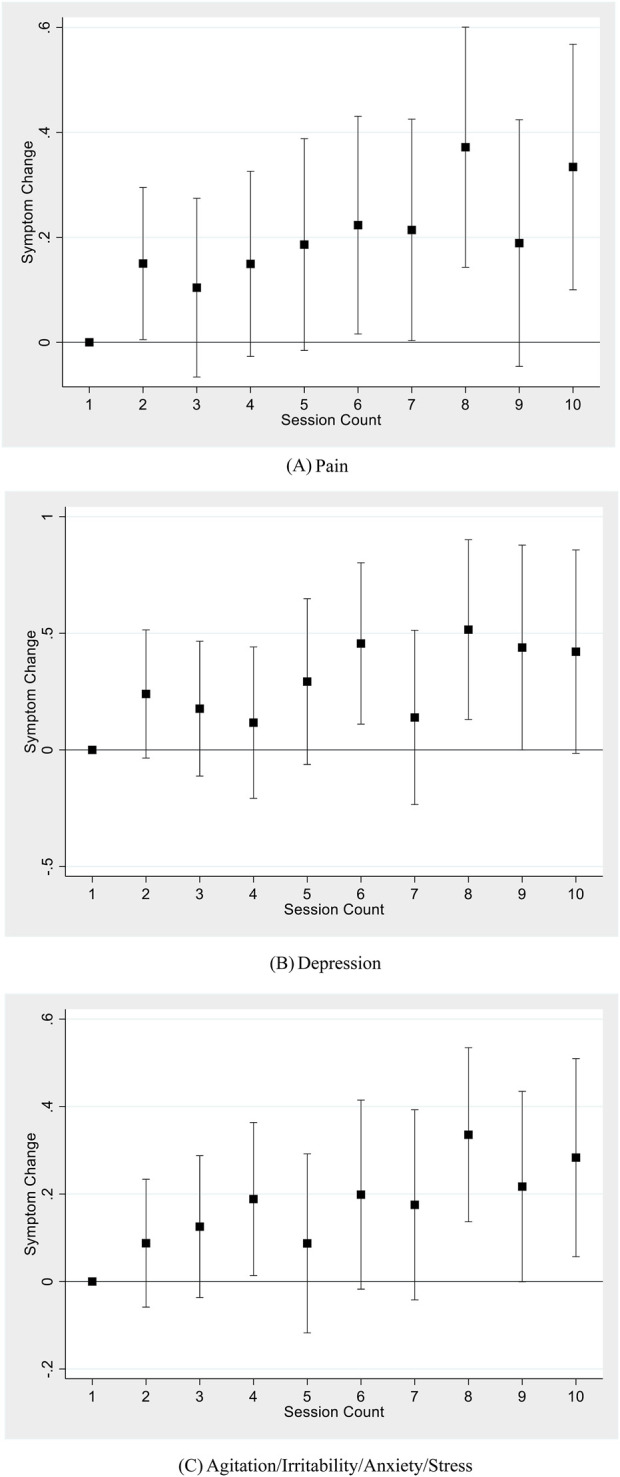
Session count and symptom change for major symptom types. **(A)** Pain. **(B)** Depression. **(C)** Agitation/Irritability/Anxiety/Stress. Notes: Each figure shows results using a sample of sessions reporting the corresponding symptom type, regressing symptom change on a set of dummy variables for each session count up to 10, including patient-level fixed effects, starting symptom level, the mode of consumption, THC and CBD potency, and natural log of dose. Standard errors are clustered at the individual level. 95% confidence bars are shown.

Table 4 reports results from subsample analyses focusing on three major symptoms: pain, depression, and anxiety (including agitation, irritability, anxiety, and stress). Results from all three major symptoms show a similar pattern: session count was associated with an increase in symptom change (a decrease in symptom relief). Pair-wise t-tests show that Table 4 coefficient estimates of session count for all three symptoms were not statistically different from each other. Figure 3 graphically illustrates the change in symptom relief as the user completes more sessions, using the nonlinear, discrete measure of session count, with the first session used as the baseline and each panel representing a different symptom. All three panels show a generally upward trend, although coefficients for the session counts in these smaller symptom-specific samples are as independently statistically significant as in the main sample.

**TABLE 4:** Subsample analyses by user experience.

	(1) Symptom change	(2) Symptom change	(3) Symptom change	(4) Symptom change
	Experienced		Not experienced	
Session count	0.026***	0.039***	0.015***	0.012
	(0.005)	(0.011)	(0.005)	(0.012)
THC		−0.007***		−0.004*
		(0.002)		(0.002)
CBD		−0.002		−0.000
		(0.002)		(0.002)
Smoke (Joint/Pipe)		−0.141		−0.138
		(0.126)		(0.172)
Eat or Drink		−0.172		−1.496**
		(0.682)		(0.672)
LnDose		−0.405***		−0.368***
		(0.054)		(0.053)
Starting symptom	−0.657***	−0.640***	−0.655***	−0.654***
	(0.006)	(0.013)	(0.007)	(0.013)
Constant	−0.001	0.902***	0.143***	0.814***
	(0.041)	(0.180)	(0.043)	(0.202)
Observations	47,070	10,346	42,900	9,786
R-squared	0.388	0.394	0.367	0.390
Number of users	5,690	1,774	4,764	1,615

Notes: The sample is restricted to those reporting their pre-app experience with cannabis. Experienced include patients reporting that they are “Expert” cannabis users or have “A Lot” of experience. Not experienced includes patients reporting that they are “Beginner” cannabis users or have only “A Little” experience. Each column represents a separate regression. All regressions include patient-level fixed effects. Smoke and eat or drink are relative to vape. Standard errors, clustered at the individual patient level, are shown in parentheses. ***p < 0.01, **p < 0.05, *p < 0.10.

### Mitigating factors: THC, CBD, and dose

Table 5 explores ways in which patients may adjust their consumption to reduce the effects of tolerance, e.g., by increasing product potency or the amount of the product consumed. Changes in THC or CBD potency would involve switching to a new product, while increasing the dose could occur with the same product. Results show that session count was not associated with increased THC or CBD level. As shown in Column 3, patients do adjust the dose consumed, increasing it by 0.6% with each subsequent session^3^. However, this is not enough to outweigh the tolerance effect documented in Table 2; a six percent increase in the dose is required to overcome the tolerance effect of another session on symptom relief.

**TABLE 5:** The association between session count and factors likely to reduce tolerance effects.

	(1) THC	(2) CBD	(3) LnDose
Session count	0.038	−0.059	0.006***
	(0.071)	(0.069)	(0.002)
Constant	36.549***	11.378***	1.387***
	(0.271)	(0.257)	(0.006)
Observations	49,022	30,800	120,586
R-squared	0.834	0.766	0.753
Number of users	7,776	5,648	16363

Notes: Each column represents a separate regression. All regressions include patient-level fixed effects. Standard errors, clustered at the individual patient level, are shown in parentheses. ***p < 0.01, **p < 0.05, *p < 0.1.

### Side effects: negative, positive and context-specific

Table 6 looks at the prevalence of side effects. Models without product characteristics and dosage are used in odd columns and models with full set of controls are used in even columns. Findings in all columns suggest that later sessions were generally associated with a decrease in the reporting of side effects, whether negative, positive, or context-specific. Product THC levels and reported dosage were associated with increased reporting of all types of side effects, just as they were positively associated with increased symptom relief. A four percentage point increase in THC would outweigh the benefits of tolerance in reducing negative side effects. A significant increase of approximately 25% would be required for the dose to outweigh the 0.004 percentage point benefit of another session in reducing negative side effects^4^.

**TABLE 6:** Subsample analyses by major symptoms.

	(1) Symptom change	(2) Symptom change	(3) Symptom change	(4) Symptom change	(5) Symptom change	(6) Symptom change
	Pain		Depression		Agitation/Irritability/Anxiety/Stress	
Session count	0.008*	0.030***	0.030***	0.046**	0.032***	0.029***
	(0.005)	(0.011)	(0.009)	(0.020)	(0.004)	(0.011)
THC		−0.006**		−0.009***		−0.005**
		(0.002)		(0.003)		(0.002)
CBD		−0.004*		−0.002		−0.000
		(0.002)		(0.004)		(0.002)
Smoke (Joint/Pipe)		−0.015		0.007		−0.273*
		(0.156)		(0.250)		(0.142)
Eat or Drink		−0.352		−2.051***		0.424
		(0.326)		(0.272)		(1.109)
LnDose		−0.367***		−0.467***		−0.450***
		(0.060)		(0.106)		(0.054)
Starting symptom	−0.618***	−0.602***	−0.706***	−0.678***	−0.725***	−0.724***
	(0.008)	(0.017)	(0.014)	(0.030)	(0.007)	(0.015)
Constant	0.194***	0.853***	0.279***	1.147***	0.080*	1.051***
	(0.049)	(0.199)	(0.088)	(0.357)	(0.042)	(0.189)
Observations	31,639	7,026	10,596	2,152	36,427	7,610
R-squared	0.327	0.334	0.424	0.452	0.445	0.477
Number of users	8,786	2,570	5,220	1,320	10,969	3,033

Notes Each column represents a separate regression. All regressions include patient-level fixed effects. Columns 1 and 2 include all patients treating a pain condition, Columns 3 and 4 restrict the sample to those treating specifically “Depression,” and Columns 5 and 6 include patients who report treating symptoms related to Agitation/Irritability, Anxiety, or Stress. Smoke and eat or drink are relative to vape. Standard errors, clustered at the individual patient level, are shown in parentheses. ***p < 0.01, **p < 0.05, *p < 0.10.

The side effects results indicate a potential silver lining from tolerance. While tolerance reduces symptom relief, it also is associated with a better negative side effect profile, which could help patients tolerate long-term treatment.

As a robustness check, we ran regressions using whether THC information is missing as an outcome variable with results reported in Supplementary Table S4. The relationship between session count and missing THC information is highly insignificant (p > 0.7). People who vape are more likely to report THC values than those who smoke or orally consume their cannabis, likely driven by the greater likelihood that vaped products are purchased rather than cultivated and because of the issue with THC reporting for edibles.

## Discussion

The current study builds on previous research highlighting the effects of using common and commercially available cannabis products for treating health disturbances (Stith et al., 2018; Cuttler et al., 2018). Using an electronic diary-method to measure real-time cannabis usage effects over the first ten sessions recorded by a patient, we observed a reduction in symptom relief and reporting of side effects (negative, positive, and context-specific) with repeated cannabis usage. This effect was robust to alternative measures allowing for nonlinear effects, using the number of days since reporting began, or restricting the sample to users completing their first ten sessions within a limited number of days. The consistency across the difference measures of cannabis exposure may be explained by the long half-life of cannabis, such that the THC level in the body a few days after consumption may not differ dramatically from that a week after the prior consumption event (Millar et al., 2018; Mørland and Bramness, 2020; Huestis et al., 1992). Observed tolerance effects were similar across major symptom categories but appeared to be significantly more evident among experienced cannabis users. THC and dose were strong predictors of improved symptom relief, as well as increased side-effect reporting. Patients did not adjust THC levels to mitigate the effect of tolerance, which could be due to access or financial constraints on trying new products or reflect a more general limitation in the range of the THC levels found in commercially available cannabis products, either way resulting in constraints on the ability of patients to explore a fuller range of product choices and treatment options. This study did find evidence that patients adjusted their dose to alleviate effects of tolerance, but adjustments were small and insufficient in magnitude to overcome the effect of tolerance. A final factor limiting the extent to which patients may be willing to adjust THC and dose to increase symptom relief is that increased THC and dose also are associated with increased negative side effects.

Tolerance to the effects of using cannabis is generally believed to result from a weakening of the functional connectivity and responsiveness of neural reward circuits that reinforce cannabis addiction—changes unobserved among novel and infrequent users (Mason et al., 2021). Cognitive functioning is among the primary domains in which people exhibit cannabis tolerance effects, although several types of physiological and psychosomatic effects of cannabis exposure have been observed to lessen after repeated usage over time (Colizzi and Bhattacharyya, 2018; Piscura et al., 2023; Ramaekers et al., 2021). Most of the previous research on cannabis tolerance effects, for example, on driving-related neurobehavioral skills and computer-based performance tasks, have focused on the role of THC (Mason et al., 2021; Sevigny, 2021) and its associations with endogenous cannabinoid receptors. Decreased cognitive reactions from repeated cannabis exposure are generally believed to result from the desensitization and depletion (internalization) of CB1 receptors from neuronal surfaces (Hirvonen et al., 2012; Burston et al., 2010; D’Souza et al., 2016). Desensitization and internalization of CB1 receptors can be returned to previous levels, thereby evidencing *tolerance reversal* (Hirvonen et al., 2012), in part through regulation of the phosphorylation of local cellular environments (Daigle T. et al., 2008) and decoupling of g-protein receptor signaling transduction (Daigle TL. et al., 2008). Downregulation of CB1 receptors and normalization of dopaminergic output is associated with reduced impairment—again, neuroadaptations unobserved in less frequent users (Ramaekers et al., 2020) and likely affected by numerous intrinsic, behavioral, pharmacological, and product-level characteristics (Ramaekers et al., 2021). Unfortunately, nearly all previous research on cannabis pharmacokinetics focused on the effects of a single, isolated phytochemical, typically the role of either THC or CBD. None of these studies, along with the current study, were designed to capture the additive and synergistic effects from multiple classes of phytochemicals commonly found in the *Cannabis* plant, including sub-cannabinoids like THCA and THCV as well as common terpenes like limonene and myrcene. Our study does improve upon prior studies using synthetic cannabinoids by using a broad range of cannabis products selected by patients but is limited by not accounting for phytochemicals beyond THC and CBD.

Future research is needed to accurately measure and test the effects of the *Cannabis* plant’s phytochemicals beyond THC and CBD. In practice, patients are left to resort to unscientific “strain” names provided by cultivators, which are used conventionally to distinguish different types of cannabis plant varieties (e.g., *C. indica* vs *C. sativa*) (Stith et al., 2019). Unfortunately, cannabis strain names are often arbitrarily labeled on product packaging and marketing materials within the medical cannabis industry and among home cultivators (Vigil et al., 2023; Bonn-Miller et al., 2017). More recent research has begun to identify and distinguish the import of cannabis chemovars (McPartland and Russo, 2001; Elzinga et al., 2015; Lewis et al., 2018; Birenboim et al., 2022), or the synergistic roles of discrete volumes and ratios of multiple phytochemicals, including cannabinoids, terpenes, and flavonoids. Some of the most common *Cannabis* plant chemovars have been shown to produce unique effects on patient outcomes, for example, demonstrating that cannabis phenotypes with high levels of terpinolene, moderate levels of THC, and low levels of CBD are more effective for alleviating anxiety and depression than the many chemovars with other terpene and cannabinoid contents (Vigil et al., 2023). It is, therefore, likely that different *Cannabis* plant chemovars not only have distinct effects across individuals, but that many more phytochemicals, beyond THC alone, affect the development and reversal of cannabis tolerance (e.g., CB1 receptor downregulation). For this reason, it is crucial that the cannabis industry promote and maintain the genetic diversity naturally arising in the cannabis plant, rather than follow the current, conventional prescription medication paradigm, whereby standardized and non-personalized medications are the only types of primary treatment options available. Indeed, a new drug evaluation paradigm may be warranted for cannabis products, because the current FDA approval process requires standardization of investigational new drugs.

Although the underlining scientific principles of how cannabis tolerance forms and can become reversed remain elusive, the results of this study have obvious and readily implementable clinical implications. First, clear tradeoffs exist between symptom relief and negative side effects. Patients can be offered hope that if they continue treatment, the side effects, such as anxiety, paranoia, and dizziness, will become less salient over time, increasing patient medication compliance. Second, the factors, including tolerance, that reduce symptom relief, also work to reduce negative side effects and *vice versa*, so that increased THC and higher doses lead to greater symptom relief but more negative side effects. If patients find the side effects of cannabis too intense, they can simply reduce THC potency or the volume of cannabis consumed. By adjusting THC levels and the volume of cannabis consumed, while taking into account the rapid onset of tolerance, clinicians can better customize cannabis therapy to specific patient needs and sensitivities, for example, by recommending varying dosing intervals and product rotation strategies.

As in most studies, there were distinct cost-benefit tradeoffs of utilizing the current observational research design. By using a mobile app to measure real-time cannabis treatment sessions, *in vivo*, the study was able to provide greater generalizability than most controlled, clinical investigations, whereby patient behaviors (e.g., dosing regimen), the products themselves, and the environments (e.g., research personnel) that enrollees experience are highly constrained by laboratory parameters (Vigil and Coulombe, 2011; Vigil and Alcock, 2014). Because the participants in the current study recorded information from the actual, commercially-available cannabis products they obtained in the retail industry, there is a strong probability that other individuals may encounter similar products, thereby increasing the current investigation’s external validity, or practical applicability to persons not directly enrolled in the research study. In contrast, cannabis’ continued Schedule I status has historically constrained most clinical investigations on the effects of using cannabis to a select quantity of cannabis samples, historically cultivated by a single governmental source, namely, the University of Mississippi (Stith and Vigil, 2016; National Academies of Sciences E and M, 2017). The results of those studies have relatively poorer generalizability, as individuals outside of the actual studies are unlikely to encounter the types of cannabis products measured in the investigations.

At the same time, the current study was limited in several ways that future research will need to address in order to better understand cannabis’ pharmacokinetics, and eventually, how best to optimize individualized cannabis treatments. While the statistically significant observation of reduced treatment effectiveness with continued cannabis use is intriguing, its long-term clinical relevance (e.g., for daily health functioning) remains unclear. Likewise, the current study was unable to incorporate several patient-level and product-level factors that are likely to influence cannabis tolerance and the treatment decisions that patients make, including physical and psychological traits, past experiences and behaviors, specific types of symptoms within broader symptom categories (e.g., neuropathic, nociplastic, or musculoskeletal pain), comorbidities, medication histories, substance use, concomitant medication usage, and more detailed cannabis product characteristics (e.g., subcannabinoid and terpene contents) that are conventionally unlabeled and/or incorrectly labeled in the current medical cannabis industry (Bonn-Miller et al., 2017; Vandrey et al., 2015). For example, changes in dosing strategies (e.g., up-titration) of other medications, or the inherent effects (e.g., either reducing or increasing symptom intensity levels) of the medications themselves may have affected the participants’ self-medication behaviors, thus confounding the interpretation of the current results. The study analyzes only the first 10 sessions of usage within a specific window of time therefore the results may not extrapolate to long-term, repeated cannabis use (e.g., after months or years). Likewise, because tolerance is largely a biochemical process that involves the actions of major and minor cannabinoids on specific (e.g., CB1 and CB2) receptors, the lack of control and qualification (e.g., via serum, blood levels) of the exact quantities and bioavailability of the consumed phytochemicals obfuscates the direct ability to measure the pharmacodynamics and pharmacokinetics of any particulate chemical metabolite, such as delta-9 THC. Finally, recall bias and/or selection bias may result from use of the mobile app if, for example, enthusiasm for using the app changes depending on one’s willingness to use the software app, or with either disappointing or satisfactory experiences from using a product. Future research will, therefore, benefit from more precise information on the set (e.g., mental context) and setting (e.g., physical context) of cannabis usage, the health status of the patient, the underlying phytochemicals in the products consumed, the immediate motivations for using cannabis, as well as the time-frame in which tolerance can be formed and reversed. Selection effects from using an online app to collect data also could influence the composition of our sample. Studies have shown that health apps are more common among females and younger individuals (Carroll et al., 2017).

## Conclusion

Cannabinoids are unlike many other medical treatments and addictive substances, because it is comprised of a multitude of chemical molecules that contribute to its cognitive and behavioral effects and these can vary significantly across plant strains and derived-products. Tolerance from the effects of cannabis may in turn be dependent on the actions of a multiple of synergistic phytochemicals, acting either additively or interactively, beyond the discrete role of any singular cannabinoid (e.g., THC) alone; tolerance reduction and perhaps even tolerance reversal may be facilitated by cannabinoid and other phytochemical rotation, and hence, by regularly switching product types. Similar to management of other medications, such as opioids, patients may also be able to adjust their cannabis treatments by changing dosing regimens, using the lowest possible dosages, and implementing multimodal symptom management. If experimentation with diverse cannabis products is effective for reducing cannabis tolerance, then a non-standardized medication model should be considered for optimizing cannabis-based treatment strategies. Certainly, more research into the complexity of the cannabis phytochemical structures in relation to symptom relief, side effects, and tolerance is needed. In the meantime, the results of this study highlight for clinicians the tradeoffs between symptom relief and negative side effects and offer readily implementable solutions for moderating the effects of cannabis tolerance through changing THC levels and dosage volumes to improve patient outcomes and treatment compliance.

## Data Availability

The data analyzed in this study was supplied from MoreBetter, Ltd. Data requests must be directed to MoreBetter, Ltd., and are available subject to approval. Further inquiries can be directed to the corresponding author.

## Footnotes

1 Abdominal, arm or leg, back, coccyx, cramping, gastrointestinal, headache, joint, menstrual, migraine, muscle, neck, nerve, other.

2 A 1% increase in the dose is associated with a $-0.386*\ln\left(1.01\right)=-0.00384$ improvement in symptom relief. 0.022/0.00384=6 .

3 Each additional session is associated a dose increase of $\exp\left(0.006\right)=1.006$ or a 0.6% increase in the number of doses in each session.

4 A 1% increase in the dose is associated with a $0.016*\ln\left(1.01\right)=0.00016$ increase in the share of negative side effects. 0.004/0.00016=25 .
