# Supplementary material for: Cannabis tolerance reduces symptom relief
Source: Front Pharmacol. 2025 Jun 19;16:1496232. doi: 10.3389/fphar.2025.1496232 (PMC12223318; doi:10.3389/fphar.2025.1496232)
Supplement: Supplementary file 1 [file Table1.docx]

Supplemental Table 1: Prevalence of side effects

| **Side Effect** | **% Sessions Reporting** | **Category** |
| --- | --- | --- |
| Anxious | 10% | Negative |
| Clumsy | 5% | Negative |
| Confused | 6% | Negative |
| Coughing | 16% | Negative |
| Dizzy | 9% | Negative |
| Dry Mouth | 27% | Negative |
| Foggy | 22% | Negative |
| Forgetful | 12% | Negative |
| Headache | 8% | Negative |
| Irritable | 8% | Negative |
| Nausea | 3% | Negative |
| Paranoid | 5% | Negative |
| Rapid Pulse | 6% | Negative |
| Red Eyes | 11% | Negative |
| Restless | 13% | Negative |
| Scattered | 20% | Negative |
| Unmotivated | 17% | Negative |
| Active | 8% | Positive |
| Chill | 50% | Positive |
| Clear | 21% | Positive |
| Comfy | 35% | Positive |
| Creative | 12% | Positive |
| Dreamy | 30% | Positive |
| Energetic | 11% | Positive |
| Focused | 23% | Positive |
| Frisky | 9% | Positive |
| Grateful | 15% | Positive |
| Great | 16% | Positive |
| Happy | 27% | Positive |
| Light | 21% | Positive |
| Optimistic | 18% | Positive |
| Peaceful | 52% | Positive |
| Productive | 13% | Positive |
| Reflective | 22% | Positive |
| Relaxed | 59% | Positive |
| Tuned | 21% | Positive |
| Couchlocked | 18% | Context-Specific |
| Distracted | 17% | Context-Specific |
| High | 40% | Context-Specific |
| Hungry | 22% | Context-Specific |
| Silly | 10% | Context-Specific |
| Sleepy | 26% | Context-Specific |
| Talkative | 11% | Context-Specific |
| Thinky | 21% | Context-Specific |
| Thirsty | 26% | Context-Specific |
| Tingly | 25% | Context-Specific |
| Visuals | 5% | Context-Specific |

Notes: Sample is restricted to sessions with a starting symptom level greater than zero and at least one symptom update within four hours. Side effects were categorized into negative, positive, and context-specific by the authors. The frequency of reporting is listed by side effect.

Supplemental Table 2: Robustness check: days since first session

|  | (1) | (2) | (3) | (4) |
| --- | --- | --- | --- | --- |
|  | Symptom change | Symptom change | Symptom change | Symptom change |
|  |  |  |  |  |
| Days since first session | 0.003*** | 0.003*** | 0.003* | 0.004** |
|  | (0.001) | (0.001) | (0.002) | (0.002) |
| THC |  |  | -0.004*** | -0.006*** |
|  |  |  | (0.001) | (0.001) |
| CBD |  |  | 0.000 | -0.000 |
|  |  |  | (0.002) | (0.002) |
| Smoke (Joint/Pipe) |  | -0.030 | -0.143** | -0.215*** |
|  |  | (0.023) | (0.0) | (0.055) |
| Eat or Drink |  | 0.118*** | -0.285 | -0.732 |
|  |  | (0.030) | (0.415) | (0.560) |
| LnDose | |  |  | -0.368*** |
|  |  |  |  | (0.030) |
| Starting symptom | -0.683*** | -0.686*** | -0.649*** | -0.646*** |
|  | (0.007) | (0.007) | (0.009) | (0.009) |
| Constant | 0.171*** | 0.177*** | 0.202** | 0.905*** |
|  | (0.042) | (0.045) | (0.097) | (0.102) |
| Observations | 193,744 | 185,083 | 42,227 | 42,226 |
| R-squared | 0.379 | 0.382 | 0.346 | 0.356 |
| Number of users | 16,395 | 15,809 | 4,808 | 4,808 |

Notes: The sample is restricted to sessions completed within 90 days of the first recorded session. Each column represents a separate regression. All regressions include patient-level fixed effects. Smoke and eat or drink are relative to vape. Standard errors, clustered at the individual patient level, are shown in parentheses. *** p<0.01, ** p<0.05, * p<0.10

Supplemental Table 3. Robustness check: Varying the days in which the first ten sessions were completed

|  | (1) | (2) | (3) | (4) | (5) |
| --- | --- | --- | --- | --- | --- |
|  | Outcome = Symptom change | | | | |
| Days to completion of 1st 10 sessions | 90 | 60 | 40 | 20 | 15 |
|  |  |  |  |  |  |
| Session count | 0.014*** | 0.012*** | 0.010*** | 0.012*** | 0.010** |
|  | (0.004) | (0.004) | (0.004) | (0.004) | (0.004) |
| Starting symptom | -0.688*** | -0.689*** | -0.691*** | -0.700*** | -0.704*** |
|  | (0.007) | (0.007) | (0.008) | (0.009) | (0.009) |
| Constant | 0.144*** | 0.151*** | 0.160*** | 0.191*** | 0.211*** |
|  | (0.047) | (0.048) | (0.050) | (0.056) | (0.059) |
| Observations | 44,640 | 41,982 | 39,300 | 31,880 | 28,983 |
| R-squared | 0.385 | 0.385 | 0.386 | 0.396 | 0.400 |
| Number of users | 2,019 | 1,902 | 1,779 | 1,461 | 1,334 |

Notes: The sample is restricted to users who completed their first ten sessions within the number of days specified in each column header. Each column represents a separate regression. All regressions include patient-level fixed effects. Standard errors, clustered at the individual patient level, are shown in parentheses. *** p<0.01, ** p<0.05, * p<0.10

Supplemental Table 4: Session count and THC non-reporting

|  | (1) | (2) |
| --- | --- | --- |
|  | THC Not Reported | THC Not Reported |
|  |  |  |
| Session Count | 0.000 | -0.000 |
|  | (0.001) | (0.001) |
| Smoke (Joint/Pipe) |  | 0.072*** |
|  |  | (0.013) |
| Eat or Drink |  | 0.510*** |
|  |  | (0.015) |
| LnDose |  | 0.007* |
|  |  | (0.004) |
| Starting Symptom | 0.000 | 0.001 |
|  | (0.001) | (0.001) |
| Constant | 0.592*** | 0.476*** |
|  | (0.005) | (0.011) |
|  |  |  |
| Observations | 120,691 | 115,126 |
| R-squared | 0.000 | 0.102 |
| Number of users | 16,395 | 15,776 |

Notes: Smoke and eat or drink are relative to vape. Each column represents a separate regression. All regressions include patient-level fixed effects. Standard errors, clustered at the individual patient level, are shown in parentheses. *** p<0.01, ** p<0.05, * p<0.10

Supplemental Figure 1: Session count and symptom change – full controls

Notes: The figure show results regressing symptom change on a set of dummy variables for each session count up to 10, including the full set of covariates, patient-level fixed effects and starting symptom level, and clustering the standard errors at the individual level. 95% confidence bars are shown.
